# Treatment of Cervical Borders

**Published:** 1899-02

**Authors:** Stafford G. Perry

**Affiliations:** New York.


ARTICLE III.

### TREATMENT OF CERVICAL BORDERS.

DR. STAFFORD G. PERRY, NEW YORK.

From whatever point it may be considered, the cervical border presents the weakest portion of every approximal cavity. It is inaccessible, it is anatomically weak, and, above all in importance, it is, from its position, most vulnerable to the insidious agents that destroy the teeth.

In considering this border, then, we have to contend with the most difficult conditions ever met with in the care of the teeth, for it goes without saying that the approximal cavities, as a class, are the most troublesome in the mouth. We can never be old enough, wise enough, patient enough, and accurate enough to care for this border too perfectly.

There is such great variety in the conditions found here that the operator is constantly meeting with difficulties that enlist his keenest interest and stimulate his highest ambition.

Logically, the first thing to consider is the preparation of this border for the reception of whatever filling material may be used. Here we meet at once with an anatomical condition that must receive most careful attention.

I refer, of course, to the increasing thinness of the enamel as the cavity extends toward the root of the tooth. Of course, we must cut until we have reached solid structure, however far we have to go; but here we are met by the question: What is solid structure? Strictly speaking, we might cut up on the root, even above the border of the enamel, and not find it.

It may be answered, "Then we had better do so." To this I assent, as a general rule, but not by any means in all, for some teeth are in a condition that a solid border could not be found without going far under the gum, and the cavity could not be filled and finished in such a manner that the gum would ever again remain perfect along this border.

I am convinced that ruthless disturbance of the gum is given little heed to by most operators—by many, who still with a separating file destroy the perfect contour, in spite of all that has been written and said against this practice during the last twenty-five years, and by others who are such radical contourists that they cannot rest until they have cut even small cavities up under the gum, and made large fillings that shall have free edges, and that, along the cervical border, almost invariably leave a slightly disturbed condition of the gum, which bleeds readily when the floss silk or toothpick, or even the brush, is used.

More than twenty years ago, in an article on the treatment of the approximate surfaces, I advocated, in some cases, this free cutting of even small cavities, but I never supposed the system would be applied generally and to teeth of good structure. I have repeatedly seen teeth from the hands of extreme contourists, where every filling on the approximal surfaces of bicuspids and molars ran far under the gum, and where that tissue at the cervical border was ready to bleed freely at the slightest touch. I will grant that such fillings are safe,—none could be safer,—but in many I have seen the teeth of such good quality that I did not consider such radical operations necessary, for the teeth would have been safe with the filling that did not reach under the gum, and there would have been no disturbance of that tissue, and, besides all this, there would have been less display of gold, and infinitely less work and pain in performing the operations. In cutting, therefore, along the cervical border I believe it to be good practice to avoid going under the gums if the teeth are of good quality, and the general conditions in the mouth are not destructive.

Then, in cutting, the diminishing enamel must be considered, and in cutting a little more, in the hope of getting a better border, there is danger of reaching such thin enamel that it may be a question if there has been any gain by this more thorough cutting. It seems to me there is no ordinary operation where nicer care and judgment are called for than in the preparation of this cervical border. It is literally a "ragged edge," which one may approach too timidly, or over which one may too boldly go.

In the preparation of it for gold there must be more thorough cutting than for any of the plastics, because the force required in condensing the gold might check and disturb an enamel border, which might be firm enough to receive a plastic. Then, too, the preparation must depend somewhat on the gold to be used.

If cohesive gold is to be used, there must be made one or two retaining points, or a retaining groove must be cut across the whole cervical border. If soft gold is selected, it may be that no retaining point or undercut will be needed.

It is probably safe to say that no dentist of experience can be found who would not be glad if he could be free from the necessity of ever making a retaining pit or cutting a retaining groove in any tooth to be filled with gold. Then would be saved the devitalization of the border of the cavity caused by cutting the fibrils, the filling of a pit or groove which is likely to be imperfectly done, and the greater danger of crushing an enamel edge which has been weakened by the making of this pit or groove. But beggars cannot be choosers, and there must be retaining grooves in every cavity to be filled with gold.

Luckily, they need not always be at the cervical border, since the introduction of the matrix, which, making the fourth wall, becomes a great help in holding the gold in place, so that the retaining pit or the groove can be dispensend with in starting the filling, and then the mallet can be used without the fear of crushing the enamel edge, and with the certainty of making a perfect adaptation of the gold.

But if the pit or groove is omitted, there must be great watchfulness lest the gold move after it has been condensed along the cervical border. I have had failures at this border years after, when I felt certain that the gold had moved slightly without my notice before I had it securely keyed in by packing against the side wall of the cavity.

In a cavity which has been prepared along the cervical border without a pit or retaining groove, a mat of soft gold can be laid, extending a little beyond the cavity, and a matrix applied, pushing the protruding gold toward the neck of the tooth, and having the effect of holding the mat in place, and making it possible to get perhaps a more perfect adaptation of the gold to the extreme enamel edge of the tooth.

If we can have both hands free, so that the gold can be held rigidly in place for the upper third of the cavity, or till there is no chance of its moving from its place, the absence of retaining pits or grooves along the cervical border may be a clear gain. This may involve the need of an attendant to hold the rubber out of the way and reflect the light, and perhaps use the mallet. If the operator prefers to use the mirror in packing the gold, or to reflect the light and select a matrix that can be fixed to the tooth, a pit or groove at the cervical border may be necessary, since the left hand is not free to hold the pieces of gold in place.

This feeling of certainty that the gold will stay exactly where it is placed is of great importance, and should make one pause in the preparation of a cavity to make mental comparison of the two systems. My own belief is that an operator should be as ready with one method as with the other.

In what I have said thus far I have in mind the use of a soft gold, such as Abbey's, Morgan & Hasting's or Rowan's soft cylinders; either of these by slight annealing becomes cohesive enough to be packed as cohesive gold, and yet without entirely loosing their soft quality.

A gold of this kind answers the purpose so perfectly that I never use a strictly cohesive gold along this border. The introduction of the de Trey as well as Steurer gold which seems to meet with much favor in some quarters, lessens the need of retaining pits, as this form of gold stays where it is placed better than any other, and can be used with less care in holding the first few pieces in place. Examination of the surfaces of fillings made of these golds shows an accurate adaptation, and it may be that they may yet prove to be most suitable along the cervical border. Any method that will encourage or allow delicacy in the treatment of this border is to be hailed as an advance. We appreciate this when we think of the days of cohesive gold and the sledge-hammer blows of the mallet.—*International.*

